# Emphysematous Pyelonephritis: Bedside Ultrasound Diagnosis in the Emergency Department

**DOI:** 10.5811/cpcem.2016.12.32714

**Published:** 2017-03-14

**Authors:** Gillian McCafferty, Amanda Shorette, Sukhdeep Singh, Gavin Budhram

**Affiliations:** * Lahey Hospital and Medical Center, Department of Emergency Medicine, Burlington, Massachusetts; † University of Massachusetts Medical School-Baystate Health Springfield Campus, Baystate Medical Center, Department of Emergency Medicine, Springfield, Massachusetts

## Abstract

Emphysematous pyelonephritis (EPN) is a rare, life-threatening infection, and misdiagnosis as uncomplicated pyelonephritis is potentially fatal. Point-of-care ultrasound (POCUS) is a valuable tool for evaluation of the kidneys in patients with septic shock and pyelonephritis. While used primarily to assess for the complication of obstruction and hydronephrosis, POCUS may also detect signs of EPN and prompt surgical consultation for nephrectomy. We present a case in which the emergency physician diagnosed EPN by POCUS in a patient with septic shock and pyelonephritis.

## INTRODUCTION

Emphysematous pyelonephritis (EPN) is a rare, life-threatening, necrotizing complication of pyelonephritis, which is usually associated with uncontrolled diabetes mellitus (DM) or ureteral obstruction.^1^,^2^ Misdiagnosis as uncomplicated pyelonephritis is potentially fatal since mortality is as high as 71–80% in those treated with antibiotics alone but improves to 20–29% with nephrectomy.^3^,^4^ In this report we describe a case of EPN that presented with abdominal pain and peritonitis and was diagnosed by point-of-care ultrasound (POCUS).

## CASE REPORT

An 84-year-old woman with diabetes, hypertension, and chronic kidney disease presented to our emergency department with four days of right flank pain with nausea, vomiting, and anorexia. She was febrile (39°C/102.2°F), tachycardic (heart rate 132), tachypneic (respiratory rate 27), and hypotensive (98/63 mmHg). On examination she was ill appearing, diaphoretic, and had tenderness in the right flank and right costophrenic angle.

**Image 1:**
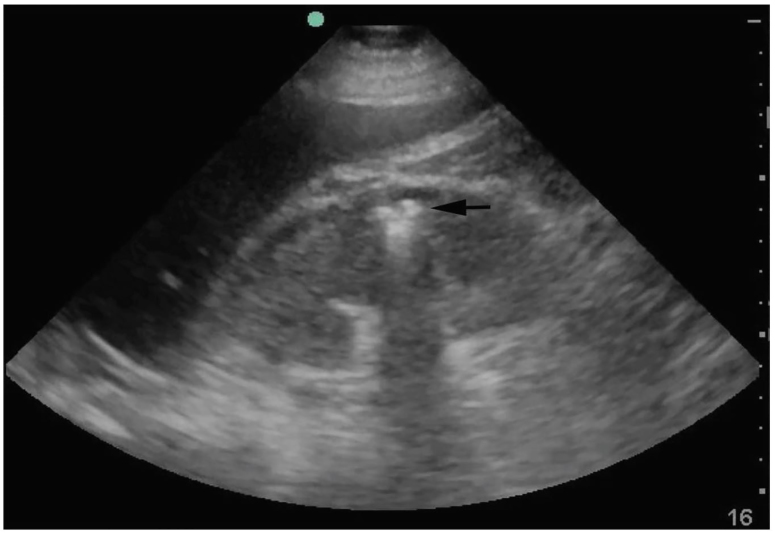
Point-of-care ultrasound demonstrating emphysematous pyelonephritis. In this still image, air can be seen in the renal cortex as a hyperechoic focus (black arrow) with posterior “dirty shadowing” as a result of reverberation artifact.

In addition to performing a rapid ultrasound in shock (RUSH) examination to assess cardiac function, fluid status and sources of shock, ultrasound showed an enlarged right kidney with echogenic foci in the renal medulla and reverberation artifacts representing air, referred to as “dirty shadowing” (Image 1). 5 This was concerning for EPN, prompting emergent urology consultation.

**Image 2:**
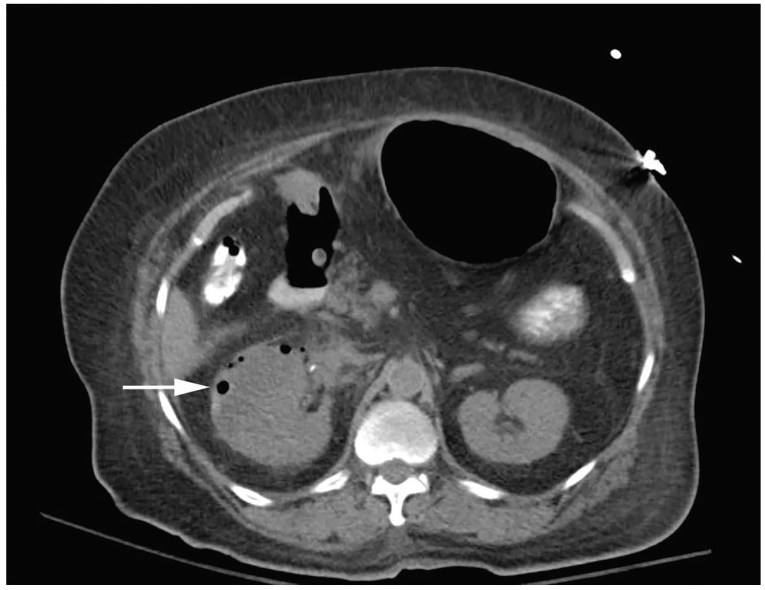
Computed tomography of the abdomen and pelvis showing an enlarged right kidney with perinephric stranding and air in the renal cortex (white arrow) consistent with emphysematous pyelonephritis.

Significant laboratory tests included a white blood cell count of 14,600/mm^3^, an anion gap acidosis with bicarbonate of 14mmol/L, lactate of 8.4mmol/L, pH 7.26, and kidney injury with creatinine 4.0mg/dL. Urinalysis showed heavy bacteria and pyuria. The computed tomography (CT) demonstrated air in the right kidney consistent with EPN (Image 2). Antibiotics were started and the consulting urologist recommended conservative management and admission to the intensive care unit.

For the next few days the patient appeared to be improving, but on hospital day six she developed altered mental status, fever, and hypotension requiring vasopressors. She was taken emergently to the operating room where an open right nephrectomy was performed. Following surgery her condition improved, renal function returned to baseline, and delirium resolved. After a short stay in a rehabilitation facility she returned home.

## DISCUSSION

Emphysematous pyelonephritis (EPN) is an acute, severe, necrotizing bacterial infection of the renal parenchyma and surrounding tissues associated with high mortality. Kelly and MacCallum reported the first case of EPN in 1898, referring to the diagnosis as pneumaturia.^6^ Initially thought to be extremely rare, the increased use of CT has resulted in more reported cases.^7^ Although described as a rare disease process, the exact incidence of EPN in recent years is poorly described.

The majority of patients have a history of diabetes mellitus. There is also a female predominance (75%), which is likely due to the higher rate of urinary tract infections in women.^8^ Urinary tract obstruction is the most frequent cause of EPN in the absence of diabetes mellitus. Ureteral obstruction was described in 50% of diabetic EPN cases and 100% of non-diabetic EPN cases.^9^

The clinical presentation is often similar to acute pyelonephritis with complaints of fever, chills, abdominal or flank pain, dysuria, nausea, vomiting, lethargy, and confusion. Laboratory findings often consist of pyuria, thrombocytopenia, and acute kidney injury.^1^,^2^ Emergency physicians (EP), however, must have a high level of suspicion for more serious acute renal infections in patients presenting with sepsis, shock, or those who have not responded to previous antibiotic treatment.

The diagnosis of EPN is most often made by CT that demonstrates air within the renal parenchyma or renal sinus.^9^ Although no studies exist that directly compare the test characteristics of CT and ultrasound for EPN, several authors have advocated POCUS as an early diagnostic tool to facilitate surgical management.^10^ In a 2011 retrospective study of 206 patients with acute pyelonephritis, 60.9% had structural abnormalities detected on POCUS. In 34.3%, the ultrasound findings effectively diverted the patient to receive surgical interventions including percutaneous nephrostomy, abscess aspiration, ureteroscopic stone manipulation, lithotripsy, or nephrectomy.^11^ In patients with suspected EPN or established diagnosis of EPN by POCUS, a non-contrast CT should be obtained to confirm the diagnosis and to provide details regarding the extent of infection.^12^–^14^

Characteristic ultrasound findings of EPN include echogenic foci in the kidney with posterior “dirty shadowing” caused by reverberation artifact from air. This should be differentiated from the posterior acoustic shadowing of intrarenal calculi, which have a distinctive echo-free shadow distal to the calculus.^15^ If fluid collections are present, EPs may note ring-down artifact from air trapped within fluid collections. In severe cases in which there is a significant amount of air or perinephric fluid, the artifact may completely obscure the kidney and make visualization difficult or even impossible.^7^,^15^

Treatment of EPN includes aggressive resuscitation, antibiotics, percutaneous drainage, or open nephrectomy. Causative organisms are most commonly *E. coli* and *K. pneumoniae*.^7^ Classic treatment of EPN involves broad spectrum antibiotics and emergent nephrectomy, although with less severe cases of EPN antibiotics and percutaneous drainage may effectively treat the infection.^16^ The overall mortality of EPN is estimated to be 25–42%.^10^,^15^ One study reports a survival rate of 29% with antibiotics alone but a 71% survival rate if both antibiotics and surgical intervention are implemented.^10^ Other studies report a mortality rate as high as 50–70% with antibiotics alone but improved mortality of 7–13.5% with antibiotics plus surgery or percutaneous drainage.^9^,^10^,^15^

## CONCLUSION

EPs using POCUS in the evaluation of critically ill patients with pyelonephritis should be aware of the sonographic appearance of emphysematous pyelonephritis. In these patients, the characteristic appearance of echogenic foci and dirty shadowing within the kidney can expedite treatment and urology consultation for nephrectomy.
